# Advanced Technologies for Enhancing Structure and Functions of Films and Coatings

**DOI:** 10.3390/foods13223604

**Published:** 2024-11-11

**Authors:** Lubomír Lapčík

**Affiliations:** Department of Foodstuff Technology, Faculty of Technology, Tomas Bata University in Zlín, Nam. T.G. Masaryka 5555, 760 01 Zlín, Czech Republic; lapcik@utb.cz

## 1. Introduction

This Special Issue, titled Advanced Technologies for Enhancing Structure and Functions of Films and Coatings, covers current trends that revolve around sustainability, multifunctionality, and the integration of smart materials. Biopolymer-based films, such as those made from gelatin, chitosan, and polysaccharides, are gaining popularity due to their biodegradability and environmental friendliness, often enhanced with bioactive substances for antimicrobial and antioxidant properties. Nanotechnology plays a crucial role, with nanofillers improving mechanical strength, barrier properties, and thermal stability. Edible coatings infused with natural extracts, like rosemary or bergamot, offer the dual benefits of protection and an extended shelf life, especially in food applications. Smart packaging that responds to environmental conditions and layered or composite films tailored for specific functions, such as improved water resistance or oxygen permeability, are also emerging. Innovative cross-linking techniques further enhance the mechanical properties of these materials, contributing to advancements in food preservation, biomedical applications, and environmentally friendly packaging solutions.

The contributions discussed in this Editorial primarily focus on the use of biopolymers, nanomaterials, and edible coatings to improve the mechanical, antioxidant, and preservation properties of food packaging and coatings. The total number of submissions to this Special Issue was eight, of which one was rejected. The contributions within this Special Issue comprise both accepted submissions and already published papers.

## 2. Enhancement of Mechanical Properties of Zein-Based Fibers

Contribution 1 explores the enhancement of mechanical properties in zein-based nanofibers through the incorporation of millet gliadin. This study reports a significant improvement in tensile strength and water stability, making millet gliadin a promising reinforcing filler for biopolymer nanofibers.

## 3. Chicken Gelatin Fibers for Food Packaging Applications

Contribution 2 discusses the production of biotechnologically derived chicken gelatin fibers using centrifugal spinning. This study highlights that chicken gelatin is a viable alternative to traditional sources, showing that it can be effectively used for food packaging, fibers, and biomedical applications due to its excellent structural properties when cross-linked.

## 4. Chitosan Coatings for Beef Preservation

Contribution 3 investigates the impact of chitosan coatings enriched with rosemary extract on beef preservation. This study’s findings suggest that these coatings can extend the shelf life of beef by reducing water losses, enhancing tenderness, and inhibiting bacterial growth, with 4% rosemary extract providing optimal results.

## 5. Antioxidant Liposomal Gel Coatings

Contribution 4 presents the preparation and antioxidant properties of phosphorylated Auricularia cornea polysaccharide liposome gels. This study reports high antioxidant activity and discusses the potential applications of these liposome gels in food preservation, as such gels retain their activity in vitro.

## 6. Coatings Reducing Browning and Enzymatic Activity of Fresh-Cut Fruit Salads

Contribution 5 evaluates the use of chitosan coatings enriched with bergamot juice powder for fresh-cut fruit salads. This study reveals that these coatings significantly reduce browning and enzymatic activity, and they extended the shelf life of the salads while improving their overall quality.

## 7. Biodegradable Polysaccharide-Based Films

Contribution 6 focuses on the characterization of bioactive polysaccharide films cross-linked with citric acid designed for food packaging applications. The films exhibit excellent barrier properties and can achieve the controlled release of bioactive components, showing promise as biodegradable packaging materials.

## 8. Polysaccharide-Based Bioactive Delivery Systems

Contribution 7 investigates the effects of an Auricularia cornea polysaccharide coating on the stability and antioxidant activity of Ginsenoside Rh2 liposomes. This study shows that this polysaccharide coating improves liposome stability and enhances antioxidant activity, suggesting it may have applications in bioactive delivery systems.

## 9. Conclusions

These papers collectively showcase cutting-edge innovations in bio-based films and coatings, emphasizing sustainability and enhanced performance in food preservation and packaging solutions.

